# What’s in a “research passport”? A collaborative autoethnography of institutional approvals in public involvement in research

**DOI:** 10.1186/s40900-016-0033-z

**Published:** 2016-06-22

**Authors:** Vito Laterza, David Evans, Rosemary Davies, Christine Donald, Cathy Rice

**Affiliations:** 1 Department of Social Anthropology, University of Oslo, Norway & Centre for African Studies, University of Cape Town, Cape Town, South Africa; 2 University of the West of England, Bristol, UK; 3 Department of Social Anthropology, University of Oslo, Eilert Sundts Hus, Moltke Moes vei 31, 0851 Oslo, Norway

**Keywords:** Patient and public involvement, Public involvement in research, Autoethnography, Complexity, Empowerment, Human resources, Reflexivity, Research relationships

## Abstract

**Plain English summary:**

The article analyses the process of securing permissions for members of the public (we refer to them as “research partners”) and academics involved in a qualitative study of public involvement in research (PIR) across eight health sciences projects in England and Wales. All researchers, including research partners, need to obtain a “research passport” from UK NHS trusts where they intend to carry out research. The article presents the experiences and observations of the authors, who all went through the process.

Research partners encountered many challenges, as the overall bureaucratic procedures proved burdensome. The effects were felt by the academics too who had to manage the whole process. This influenced the way research partners and academics built social and personal relationships required for the successful conduct of the project. We also discuss the tensions that emerged around the issue of whether research partners should be treated as a professional category on their own, and other issues that influenced the PIR processes.

In the concluding section, we make a number of practical recommendations. Project teams should allow enough time to go through all the hurdles and steps required for institutional permissions, and should plan in advance for the right amount of time and capacity needed from project leaders and administrators. Bureaucratic and organisational processes involved in PIR can sometimes produce unanticipated and unwanted negative effects on research partners. Our final recommendation to policy makers is to focus their efforts on making PIR bureaucracy more inclusive and ultimately more democratic.

**Abstract:**

**Background**

In the growing literature on public involvement in research (PIR), very few works analyse PIR organizational and institutional dimensions in depth. We explore the complex interactions of PIR with institutions and bureaucratic procedures, with a focus on the process of securing institutional permissions for members of the public (we refer to them as “research partners”) and academics involved in health research.

**Methods**

We employ a collaborative autoethnographic approach to describe the process of validating “research passports” required by UK NHS trusts, and the individual experiences of the authors who went through this journey – research partners and academics involved in a qualitative study of PIR across eight health sciences projects in England and Wales.

**Results**

Our findings show that research partners encountered many challenges, as the overall bureaucratic procedures and the emotional work required to deal with them proved burdensome. The effects were felt by the academics too who had to manage the whole process at an early stage of team building in the project. Our thematic discussion focuses on two additional themes: the emerging tensions around professionalisation of research partners, and the reflexive effects on PIR processes.

**Conclusions**

In the concluding section, we make a number of practical recommendations. Project teams should allow enough time to go through all the hurdles and steps required for institutional permissions, and should plan in advance for the right amount of time and capacity needed from project leaders and administrators. Our findings are a reminder that the bureaucratic and organisational structures involved in PIR can sometimes produce unanticipated and unwanted negative effects on research partners, hence affecting the overall quality and effectiveness of PIR. Our final recommendation to policy makers is to focus their efforts on making PIR bureaucracy more inclusive and ultimately more democratic.

Public involvement in research (hereafter “PIR”) has become a prominent feature of health sciences in the UK in the last 20 years, and it is now a requirement from many major funders of health research [24], in particular the National Institute for Health Research (NIHR). Involvement in research is often not well conceptualised [5, 6] but the most common definition used is that of INVOLVE: “an active partnership between the public and researchers in the research process, rather than the use of people as the ‘subjects’ of research” [24]. Members of the public from different backgrounds now sit on team meetings and management committees alongside academic researchers, and are involved in a variety of research activities, from informal advising on the development of interview protocols, questionnaires and information packs for participants, to carrying out data collection activities. Academic studies on public involvement have grown accordingly. There are a number of studies of involvement in different sectors of health research (e.g. [10, 26, 34]), and several major reviews of the literature on PIR have been recently produced [5, 6, 12, 21, 43, 44]. While PIR is particularly advanced in Britain, there is growing interest in other countries too, especially in Australia, New Zealand and North America [42], making the discussion in the British context relevant to a wider international audience of health researchers, policy makers and interested citizens.

Despite this surge in interest, we still lack a systematic understanding of PIR as an organisational process integral to health research, and of the implications of involving members of the public in terms of adaptation and modification of current practices in the host institutions and the health services organisations involved in research projects. There are numerous works that emphasise the importance of devoting extra resources, both in financial and time/workload terms, to enhance the chances of meaningful public involvement (e.g. [4–6]), but there are very few works that primarily focus on the socio-technical and organisational aspects of integrating members of the public in the research team. Martin and Finn [28], for instance, stress the importance of understanding processes of public involvement from an organisational perspective that takes into account teamwork, the complex institutional interfaces of health systems, and the enablers and barriers of integration of members of the public in these contexts. However, much of the literature on PIR (e.g. [33, 41]) tends to lack an explicit and systematic treatment of the specificities of the organisational and institutional context that characterise health research in the UK. The latter is influenced by distinctive and complex processes of institutionalisation, permissions, partnership with multiple stakeholders, collaboration with industry and government organisations, that set it apart from other fields of research. The literature rarely touches upon these issues and their implications for meaningful public involvement (noticeable exceptions are [47, 50]).

We address some of these challenges by focusing on the experiences and reflections of the authors – three members of the public involved in research (hereafter “research partners”) and two academics in a multi-institutional qualitative health research project – as they went through the complex process of securing institutional approvals for “research passports” from the host university and the local National Health Service (NHS) trusts to carry out data collection in specific sites. The research passport is a form with different sections to be filled in by the institution to which the research partners are affiliated, and NHS trusts in England and Wales. The analysis presented here shows important interfaces between the process of securing institutional permissions and wider organisational processes surrounding PIR both at project and institutional levels. These issues are addressed in the wider context of UK health research emerging trends, which is worth exploring further, especially in relation to its interactions with barriers to and enablers of meaningful public involvement.

## The project

The authors were team members of the NIHR-funded project “Public involvement: Assessing impact through a realist evaluation”, a partnership of the University of the West of England with Coventry University, led by principal investigator David Evans (one of the authors). The project was a realist evaluation [36–38] of PIR across eight case studies in England and Wales, focusing on the relationships between the context (e.g. the history of institutional support for involvement in a research organisation), the mechanisms that support public involvement (e.g. training or mentoring) and the desired effects or outcomes (e.g. improved research design). The general aim was to identify regularities of context, mechanism and outcome (CMO) in PIR. The eight case studies presented a diversity in terms of the people involved (young people and families with children as well as adults), the forms of public involvement and the type and scale of the research. The sample included a clinical trial, qualitative research, and studies in diverse areas of health policy and research ranging from rheumatology to mental health. The project itself featured public involvement from its early stage. Four research partners were already involved at the design and bid submission stage and included as co-applicants. Two young people were recruited as research partners at a later stage in the project, bringing the total number of research partners in the team to six. Research partners were involved in all stages of the project lifecycle, including carrying out data collection in the case studies. Data collection activities primarily involved semi-structured interviews, group observations and informal group discussions. Research partners were active members of the team and participated in all team meetings, design and implementation of data collection tools and other project activities, presentation of findings at workshops and conferences, and the production of written outputs, including the final report and other publications [14].

Only four of the eight case studies required securing of institutional approvals from local NHS trusts, as the other four case studies were university or third sector based and so did not require research passports. Only three of the six research partners on the team were involved in case studies where such permissions were required, and they are all authors of this paper: Rosemary Davies, Christine Donald and Cathy Rice. The other two authors are the academics who facilitated and supported the securing of institutional approvals for research partners and other academic members of the team. Vito Laterza, as lead researcher and project coordinator, was involved in all the aspects of this process, working in close collaboration with the principal investigator, David Evans. Another academic member of the team, who was not involved in the development and writing of the article, provided substantial support to one of the research partners throughout the research passports journey. The main reason why this academic member was not involved in our study was that, at the time, her workload did not allow for further commitments.

## Methodology

In this article, we reflect upon the processes of securing institutional permissions for research partners to carry out data collection. One thing that clearly emerged from our conversations is how different the individual experiences of the people involved were. Some of these differences, as we will explore more in depth in the discussion section, were related to the differences in roles between academics and research partners; but there were many other differences that were individual and cross-cut the research partner/academic researcher distinction. The primary aim of this reflective process is to present these multiple trajectories and voices, while at the same time highlighting emerging common themes. For this reason, and in order to emphasise the narrative aspect of our experiences, we chose to carry out the research for this article as a collaborative autoethnography. According to Chang, Ngunjiri and Hernandez, this is “a qualitative research method that is simultaneously collaborative, autobiographical and ethnographic” ([8], p.17).

Ethnography is the core method of anthropological research, and in recent decades has been widely used by other social science disciplines too – especially sociology and human geography. It is now frequently used in health science as well. The original formulation of this method emerged from the anthropological study of non-Western societies, and was consolidated in the first half of the 20^th^ century. It presents several features that set it apart from other qualitative methods. The ethnographer spends long periods of fieldwork in the community under study - often more than one year per project - to gain a finely grained understanding of everyday life and structural factors in that community. While the primary research focus remains specific – for instance, the experience of pain among hospitalised young people in Bristol – the ethnographer tries to look at the selected topics in connection with a wide range of social domains. To continue with the example of the Bristol pain study, the ethnographer would look at how young people in the study relate to their families, how their hospitalisation and experience of pain affect their studies or work life, how the labour market environment and local and national government affect their life prospects and well-being, and so on and so forth.

This is another way to say that the ethnographic method is holistic. The boundaries of the study are not set in advance. An exploratory approach is used, to allow for the open-ended gathering of knowledge on the real linkages and connections in the social reality under examination, rather than the imposition of a theoretical framework driven by the ethnographer’s own assumptions about the topic of study. A variety of qualitative – and sometimes quantitative – research methods drawn from other social sciences are used, including interviews, collective discussions, questionnaires, and observations. One research activity that is distinctive of ethnography is “participant observation”. The ethnographer participates in various social events and situations that make up the everyday life of research participants; at the same time, the ethnographer thinks and writes about his/her participation as a way to gather knowledge about the community under study.

In this sense, ethnography is inherently reflexive. “Reflexivity” is a widely used term in qualitative social sciences and social theory, and in this case refers to the ability of the ethnographer to see him-/herself as part of the research context. He/she is not an external observer of independent processes of cause and effect. Rather, his/her presence and methods influence the reality under study and the findings. The relationship between researcher and research participants becomes an important part of the study. Our study adopts an ethnographic orientation, in line with the principles highlighted above. These are general guidelines, rather than fixed prescriptions, and need to be adapted to the specific context within which one operates.^1^

What is a “collaborative autoethnography”? An autoethnography is a kind of ethnographic research where the researcher not only participates in the life of research participants, but also shares key experiences and social identities with the research participants, and decides to include his/her own account alongside that of others in the study. To carry on with our previous example, if the ethnographer is a young person from Bristol with experience of pain and hospitalisation, and includes him-/herself in the study, he/she is carrying out an autoethnography. One important feature of autoethnography is the dual role of the ethnographer both as participant in the processes under study, and detached observer of their participation. This further enhances the reflexive dimension, enabling the extraction of abstract knowledge about the particularities of events, processes and contexts in a way that other more standardised methods might lack.

In our case, all the academics and research partners involved in the study reflected about their own experience of the research passports journey. Like autoethnography, our study focuses on “self-interrogation, but it does so collectively and cooperatively within a team of researchers” ([8], p. 21, in [30], p. 6). Individual moments of research and reflection are alternated with collective activities.

As these meetings and interviews took place within the structures of the project, the methodology developed embodies some of the key aspects of everyday interaction and collaboration between academics and research partners in the project as a whole, marking the kind of “everydayness” that characterises ethnographic studies. The open-endedness and reflexive dimensions set our study somewhat apart from more structured approaches in evaluation where collective discussions also play an important role (see for instance [2]). We are not claiming that our method is better than other more established qualitative methods to measure impact. Rather, we argue for the need to have a plurality of approaches to understand and evaluate different aspects of public involvement. We felt that the specificities of the experiences and processes we want to address called for the approach employed here.

This open-ended process stimulated deep and intense exchange about other issues in the project (e.g. the limitations of NHS research governance procedures as applied to ethnographic methods). Although we can only touch upon some of these issues in passing here, this shows the value (and the challenge) of adopting a reflexive methodology to explore complex institutional processes which involve multiple stakeholders, organisations and societal and policy discourses. The ethnographic orientation enabled us to focus on certain emergent themes without losing sight of the broader context. These other relations should not be obscured, hence references to these wider issues are made throughout.

Finally, we chose this approach because we believe that reflexive ethnographic methods are well suited for a more participatory approach where participant observers from different backgrounds (e.g. research partners and academics) lead the process beyond the formal boundaries of the interview, and emphasise different aspects of the reality under analysis without being too tied to a pre-established set of questions and topics.^2^ The challenge here is to strike a balance between the need to present research processes in a clear and accessible fashion to research partners, and the aspiration for openness and an exploratory approach that privileges participants’ experiences over a rigid set of specific questions.

**Fig. 1:**
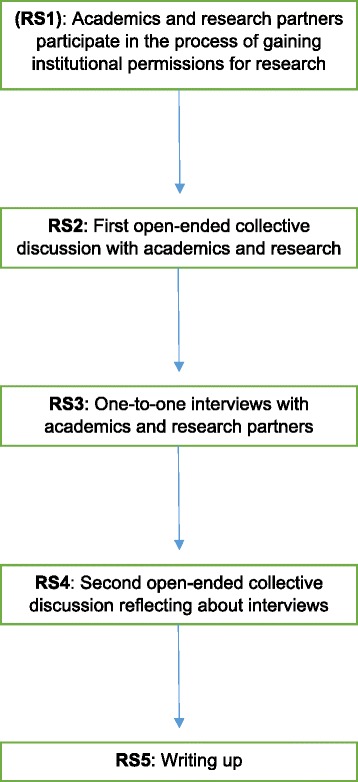
Research process flowchart. RS stands for “research stage”

We inserted a flowchart (Fig. 1) to highlight the succession of the main research stages. The first one (RS1, Fig. 1) is the participation of academics and research partners in the research passports journey. We put this step in brackets to indicate the fact that, at the time, we were not actively reflecting upon it for the purposes of this study. We started the formal research process with a collective discussion in early May 2012 (RS2, Fig. 1). On this occasion, we discussed the methodology and how the research would unfold. We also discussed some of the emerging themes for further elaboration and discussion in the paper. It might seem counterintuitive that we discussed some of the emerging themes in the collective discussion before the interviews, but the experience of going through the research passports was already there as raw data to inform the first collective discussion. There is a retrospective element to our study. However, by the time we sat down for the first collective discussion, our experiences were still fresh; our memories were also partly inscribed in the significant amounts of emails and documents that accompanied the research passports journey.

We then carried out five one-to-one interviews with each other (RS3, Fig. 1), and chose interviewer-interviewee pairs in a rotational pattern (e.g. A interviewed B, B interviewed C …) with the effect that academics were interviewed by research partners and vice versa.^3^ This contributed to a positive subversion of conventional roles in academic research, levelling out some of the hidden hierarchies and assumptions of professionalisation guiding health science research teams. The interviews were carried out between May and June 2012.

While we did not have a structured interview protocol, we devised general guidelines for the interviewer. The main goal of the interview was to capture the interviewee’s perspective on the process of securing research passports, including a detailed narrative of personal involvement with individual and institutional contacts, highlighting specific experiences that particularly affected or shaped the person’s perception of his/her role in the team and the project, and asking the person to reflect more abstractly about his/ her own experience and the implications for involving research partners in health science research. In early July 2012, after all authors had listened to the interviews, we had another collective discussion (RS4, Fig. 1) to finalise the emerging themes and plan the write-up (RS5, Fig. 1).

Vito, one of the academics involved, took the lead in two ways. As the collective discussions took place, he wrote on a board the emerging themes and key points emerging from the discussions. He also took the lead in the writing up of the paper and the further analytical work required to structure the themes and findings agreed in the final collective discussion. This division of labour was informed by pragmatic needs, primarily the time constraints under which both academics and research partners were working (see also [2], p. 611). All stages of the write-up were scrutinised by all the co-authors, and received their final approval. Co-authors contributed with suggestions for edits, modifications and deletions throughout.

We discussed the issue of ethical approvals. We came to the conclusion that we did not need ethical approval for a number of reasons. First, we agreed to offer our own individual experiences and reflections, not so much as subjects of study, but as primary drivers of the research – in other words, verbal informed consent to continue with this was deemed as sufficient, as we all agreed to collectively analyse each other’s experiences. Secondly, no data from other individuals was included. Third, the inclusion of details of relevant institutions took place in the framework of the authors’ reflections and own judgement about processes which are central to the values, principles and activities underpinning public involvement in research. We felt that, both as academic experts and research partners drawing on lay knowledge, we had the academic freedom to provide our commentary and sensitive judgements on these important matters without the need to undergo further ethical scrutiny at the university or other institutional levels.

We agreed that we did not want to reduce our multi-faceted personal experiences to a single unifying narrative. We decided to structure the article to suit this multiplicity of voices. We first present the actual process of securing research passports to give a sense of the complexities and challenges encountered. We then present individual vignettes of the single participants/authors of the article, stressing the main themes that characterise their individual experience. In the final thematic discussion, we tie the emerging themes together and synthesise them.

### The research passports process

The process of securing institutional permissions for research partners so they could carry out data collection revolved around the validation of a “research passport”. Multiple signatures and checks requiring additional documentation were needed to complete the form which then became the “passport”. There were further procedures for other NHS trusts not involved in the validation of the passport, before they could provide “letters of access” allowing research partners to carry out research in NHS sites. On paper, the same process was carried out for academics who needed a research passport. In practice, the hurdles and challenges for research partners made it considerably different from the process academics went through, as we will explore further throughout the discussion.

The first level of validation occurred within the researchers’ own institution.^4^ After filling in the relevant sections of the form, the applicant needed to meet with a manager in Human Resources (HR) assigned for this purpose. Most of the accompanying documentation required by the NHS trusts needed to be prepared for the HR manager’s check. This included:

- a Curriculum Vitae (CV);
- an occupational health screening certificate;
- a Criminal Records Bureau (CRB) (in our case, a standard CRB as there were no regulated activities in our project);
- a Good Clinical Practice certificate.

A further layer of complexity was added by the fact that the status of research partners in the institution as employees was ambiguous. While there had been a significant tradition of PIR in the centre hosting the project, this was the first project that required such levels of permissions. Other projects in the NHS were categorised as service evaluations, hence not requiring research passports, or different projects not carried out in NHS sites. While research partners were paid for their time through casual payroll claims, they did not have files with the HR service as full-time or part-time employees on contract. It took some negotiation from the project team with the HR department to develop a procedure that would enable research partners to participate in the process without undue burden or exclusionary measures, while at the same time satisfying institutional requirements. It became clear at this stage that the whole process implied, without ever making it explicit, that its main intended recipients were professional researchers or other professional staff, who were exposed on a daily basis to similar routine procedures and were usually versed in understanding the general logic of such systems. Hiccups could occur for professionals too, yet research partners on the team found on the whole that the process was not particularly attuned to their needs and diverse backgrounds. While professional researchers were used to “ticking the boxes” when it came to forms and procedural requirements, research partners felt somewhat under scrutiny as if their status and skill in the project were directly questioned by the requirements imposed upon them.

So for instance producing a CV in a professional format was not such an obvious step for some of the research partners, and took some time and advice from academics on the team. References were requested by the university HR department in order to standardise the processing of research partners’ passports in a similar way to university employees. This was relatively straightforward as academics on the team could easily provide references – many of them had worked on previous projects with the research partners. However, the meeting with the HR department also involved checking any gap in employment. While this was a standard procedure for professionals, asking such a question to research partners could be problematic. They did not enter projects as professionals, or had not in most cases followed conventional routes of professionalisation as researchers. All research partners reported that the question was not posed in a threatening way, yet it was a potentially sensitive question. It required research partners to justify long breaks in employment often due to illness or other adversities.

The occupational health screening certificate presented some sensitivities for research partners, who might have been uncomfortable with disclosing a major illness which, in some cases, was the main reason for their involvement as service users. The Good Clinical Practice certificate was taken as an online exam by two research partners, and proved to be a rather complicated process. The knowledge acquired was important to understand the procedures in place to ensure safe and robust research in clinical trials, but seemed to be less relevant for our research, which was qualitative and engaged with a diverse range of case studies. Only two case studies out of eight were clinical trials, and even in these cases, we felt that the knowledge acquired was not necessary for the kind of research interactions we had in the field. For another research partner it took some time to find an appropriate venue for the day workshop, and that too constituted a further burden on her and on the academics who helped her with booking.

Another challenge was the lack of standardisation across the different NHS trusts. The documents listed above were required by all three NHS trusts involved in our project, but there were additional forms required by some trusts that were not required by others. The processing time for validation varied greatly, and each trust had its own contacts and internal procedures we had to comply with, putting further strain on our team. Although we did attempt to standardise as much as we could the whole procedure by sending detailed instructions on most steps to the research partners, it became clear that how each step was to be implemented in practice, and how long it would take, were not things that we could easily predict or be certain about. This is not uncharacteristic of routine institutional processes. However, in this case there was a general sense from research partners that the uncertainty produced anxiety and discomfort, at a crucial stage in the project when they were trying to navigate the complex processes of doing research in an academic environment. This happened at the early stage of the project lifecycle when the team was trying to develop its own practices and work routines, and added a further layer of complexity to the demands of starting data collection across the case studies. We will now turn to the individual accounts of the experience of securing a research passport. We will refer to the authors with their first names. Rosemary Davies is known as Rosie, and Christine Donald goes by Chris.

### ROSIE

Rosie was an experienced middle-aged research partner from a mental health background, with over ten years of experience of involvement in research, including user-led research. Like the other research partners, Rosie found the research passports process quite burdensome. In her case, there was a further complication as she was also carrying out her own PhD on PIR, based at the university hosting our project. Because of this, she started the procedures to acquire a research passport before other team members. As a PhD student, she had to go through different institutional contacts who would validate the relevant sections, rather than going through the HR procedure followed by the others. She secured a research passport before anybody else in the team (including the academics) and for this reason she did not benefit from detailed guidance circulated by Vito and David. As Rosie put it, “nobody was unhelpful or hostile, but it still felt like I was forging a new path, like some primeval jungle.”

In her double role of research partner and PhD student, AUTHOR3 was particularly concerned with the pervasiveness of bureaucracy in health research, and the potential adverse effects this could have on attracting and integrating research partners into research teams. She felt that while academics might have gotten used to these processes, “form filling” was not something that took into account the diversity of backgrounds research partners might come from. The following quote well captures her concerns:

It just does feel that there is so much bureaucracy around research really, so much form filling, so much signing things to say that you guarantee this or you will do that, on the forms in a way you sign your life away, you say “yes you will do this according to this”, “you will make sure it’s ethical”, “you’ll do this, you’ll do that”, so it feels like quite a formal bureaucratic process, which actually feels quite intimidating, I think it’s quite an intimidating process. Maybe it doesn’t feel intimidating when you are a professor and you are used to doing it, but even then I don’t think anybody likes doing the forms. It’s quite a struggle.

Rosie felt that the bureaucratic requirements worked as a kind of “test” on research partners, who were tacitly expected to have some of the skills of professional researchers to be able to complete all the stages of the application. She added that this could act as a barrier for research partners who might not have the skills required, further endangering the inclusion of research partners from diverse backgrounds in health research projects.

Because of the challenges posed by bureaucratic procedures, Rosie stressed the importance of the right support structures being in place to assist research partners throughout the process. It was important to her that research partners understood from a very early stage the complex nature of this procedure, so that they could make an informed choice of whether they wanted to continue with it.

### CATHY

Cathy was a newer research partner than Rosie. Also in her middle years, she had been involved with several smaller research projects in the university following her experience of stroke at a relatively young age. If Rosie’s main feeling throughout her interview was frustration at the complex procedures she had to go through, Cathy talked openly about being deeply irritated by merely thinking about the research passports journey some weeks after it had ended. Cathy started the interview by saying that “thinking about this whole process has made me really grumpy.” This clearly shows the potential adverse emotional effects of bureaucratic procedures on research partners.

Despite Vito drafting an ad hoc guide for research partners, Cathy felt that it was still not enough to avoid major hurdles and stress at different stages of the process. She saw herself as the kind of person able to cope with forms, and yet she found the whole procedure quite complicated. The only step that worked out better than expected was the CV: she was told initially that it had to be presented in a specific template, and it later turned out that that requirement was waived.

Cathy’s main concern was that each step required individual trips to different sites in the university and other NHS trusts, and each visit had to be negotiated to fit the availability of both parties. She had to go to the university research office to start the CRB request; then she had to go to the HR department at a different site to show all of the required documents so that the university section of the research passport could be validated. Next, she had to show the same documents to someone at the research office of an NHS trust for further validation. Finally, as a holder of a non-EU passport, she had to go to the NHS trust HR department, at another site before she finally gained a letter of access a few days later. The whole process took eight weeks despite Cathy doing everything she could to speed up procedures. Cathy’s sense of frustration is well captured by the following quote:

It wasn’t that any single thing was hard, or that it was an unreasonable thing to ask, it was just one after another after another, and…each of them involved making appointments with people.

She felt that academics had it easier this way, as their full-time involvement with the institution meant that they could more easily take time off for these visits within their working hours, and it did not take the same amount of preparation and travel time to achieve this goal. The extra time and effort required for these steps might have easily put off some research partners who had multiple commitments and might not have been able to find the necessary time to go through all these hurdles.

In addition to the practicalities of arranging these trips, Cathy stressed that research partners did not have a deep knowledge of the institutional structures due to their limited time involvement, hence arranging each of these visits and appointments presented an extra effort of not really knowing what the informal procedures were about contacting somebody, how long they were expected to wait before chasing somebody for a reply, and other similar issues. Here too, it was easier for academics to negotiate their way around the institution as they were more fully integrated in it.

### CHRIS

An older research partner, Chris had been involved in a previous university-based project as a peer researcher interviewing other older people. Like the other research partners, Chris stressed the challenges of going through the complex process of securing a research passport. Like Cathy, she also felt that despite the guidance provided, each step involved certain complexities that were not clearly highlighted in advance, and needed to be dealt with ad hoc, trying to find a way into filling all the requirements. Chris too stressed the extra time required to arrange and attend different appointments. Chris’ concern was further strengthened by the fact that she lived in Gloucestershire, more than an hour drive away from the university and the NHS trusts.

In Chris’ account, one factor clearly emerges: she highlighted how her close relationship with another academic on the team, Debbie,^5^ who assisted her, was crucial for her to get through the process without feeling overburdened or isolated. Chris had worked with Debbie on previous projects, and in this project they worked in partnership to conduct data collection and analysis in one of the case studies. For Chris, trust between researchers and research partners, and the quality of the relationship between them, were crucial to overcome the challenges of complex institutional procedures:

If the foundation is there, then because you are going through these hurdles with a person that you trust, a person who has your strength in similar but also in different ways, you are willing to do whatever is required…The other side of the coin is: if I was with somebody that I didn’t know, that I never worked with before, that didn’t know me, then those things would have been much more difficult.

Chris was aware that one cannot neatly separate between the research passports journey and other team processes. The experience of going through the research passports took place while other team practices were also evolving and solidifying. A lesson to draw from this is that the effects of the research passports process on teamwork should not be underestimated.

Chris stressed another positive factor of the experience: as a result of her engagement with complex institutional procedures, she understood the general context of health research much better, and this enabled her to contribute more meaningfully to the project.

### DAVID

David was an established middle-aged academic who for several years had taken a lead in his university and more widely in seeking to promote good practice in public involvement in research. As the principal investigator of the project, David was involved in all stages of the process. His concern was that everything run smoothly enough to enable research partners to secure the needed permissions, while not overburdening them. At the same time, he had to manage multiple interactions and relations with different individuals and institutional contacts, both in the university and in the NHS trusts, in a way that constructive relationships were maintained throughout. David mentioned the emotional challenge of having to manage these multiple relationships, sometimes mediating among divergent interests, and the potential emotional distress of research partners and other academics involved. David’s concerns highlighted another dimension of the emotional work involved in such processes:

When I reflect upon this, what I’m very aware of is both the emotional work and practical work for me and Vito in all this. I think it all goes back to the fact that the university is a large institution and like all big institutions, it has a series of rules and procedures that everybody follows. And service user research partners don’t fit into that. So we had in this bit of the university over the last five or six years an approach to working with service users as research partners and paying them, which is not formally recognised by any of the university’s rules or systems, so the way I’ve always described it is that we operate “under the radar”. The thing that is interesting about this episode about research passports is that it’s forced it “above the radar”. And that meant lots of challenges and learning.

For David, one of the major challenges was that research partners did not have a clear status within the institution. This created a number of hurdles as the institutional processes required formalisation for the purposes of the research passport. In the past, involvement of research partners in the centre hosting the project did not require such level of institutional permission, hence complex formal procedures could be avoided. The lack of formalisation on the whole had benefited good informal relationships between research partners and academics. But in this case, David needed to use his informal relationships with HR staff and senior managers to secure agreement to a formal procedure that had not been in place before (on the essential role informality plays in making formal procedures work, see also [23]). David had to overcome some discomfort on the HR side in applying professional procedures reserved for university employees to research partners occupying an ambiguous position in the university personnel structures.

David emphasised that another important factor that made our situation distinctive was that research partners in our project were involved in data collection, a rare occurrence in other health sciences projects with a PIR component. The passport was required exactly because of the research partners’ deep involvement in research.

An additional major challenge highlighted by David was the need to adapt to different procedures that were not always clearly formalised or standardised. This posed problems for the future as well, because institutional procedures were likely to change in the medium-term, and different trusts presented differences in their procedures too. Despite the challenges, David thought that this constituted a good learning experience for the team for future projects. In the future he would make sure that the level of support and resources devoted to this process would be better assessed in advance.

David stated that some of the challenges encountered by research partners were also shared by academics going through the same process. However, he concluded that research partners encountered different kinds of challenges and overall for them the journey was significantly more burdensome.

### VITO

In his late twenties at the time of the project, Vito was an early career anthropologist with a substantial experience of collaboration and knowledge coproduction with members of the public during his PhD research on the anthropology of labour in Swaziland. This was his first experience with PIR in health research. As a full-time project research fellow and project coordinator, Vito worked closely with David in all stages of the process. The research passports process started about one month after he took up his post, and constituted his main activity for some months, together with setting up data collection in the case studies. For both Vito and David, this was the first time going through the research passports procedure.

Vito felt that, quite quickly into his post, the balance of his work time shifted from research to administration and coordination tasks. This was in great part due to the research passports process and the time and effort required to manage all its aspects. As this happened early into his involvement with the project, it affected Vito’s perception of his role shifting from a primarily research role to a primarily admin and coordination role. While he got used to the complex bureaucratic procedures quickly, this negatively impacted on his expectation that his primary focus should have been research, and not project management.

Despite this, Vito found the process a good entry into understanding the complex workings of health sciences in the UK. It stimulated his intellectual curiosity and made the experience worthwhile:

As an anthropologist I found the process fascinating, even though frustrating at times, it made me understand what this health science bureaucracy consists of, also because it is a bureaucracy at the interface of many other fields, it is a very good case study…, you get managers, you get people in the NHS, it’s not just researchers…all the pain and suffering we had to go through in different ways, that’s what anthropology is also about,…when you have a bit of time to reflect, you realise that they really shaped the way you understand the world.

As an anthropologist interested in organisational systems and their socio-political context, Vito found the whole process emblematic of changes in the wider society leading towards an increasing bureaucratisation of tasks and a pervasive presence of “audit cultures” (e.g. [32, 46]). This posed serious questions about the balance of time spent in projects between governance and institutional processes on one hand, and the actual research and data collection and analysis on the other.

Another effect of his involvement was that, while he was initially interested in pursuing ethnographic research in hospitals after the end of the project, he now felt that the institutional permissions required to do more exploratory kinds of research, together with the ethics committee procedures, would ultimately make it very difficult for him to pursue the project he wanted. There were other considerations that led to this, but the research passports journey played a major role in discouraging him from continuing with health research in NHS settings. He felt that other researchers from diverse academic backgrounds might feel the same way and these processes might unintentionally work as barriers for less conventional kinds of research to be carried out in health sciences.

### Final thematic discussion

In the final collective discussion we held after listening to the individual interviews, we discussed the similarities and differences between our individual narratives and some of the emerging themes. This section is for the most part inspired by that collective conversation.

What emerges from all the accounts is that the overall bureaucratic procedures of securing a research passport proved to be burdensome for research partners. The process was not an easy one to manage for the academics involved either. What proved to be much more challenging than expected was the emotional work required by all of us. A further challenge was that this took place while we were forging interpersonal relationships that would prove crucial for the successful conduct of the project.

In the end, all research partners involved obtained the required permissions to conduct research in their case studies. This shows that, despite the hurdles, the procedures were flexible enough to allow people from different backgrounds who were not professional researchers to obtain the research passport. It additionally shows that the relationships between the academic researchers and the research partners were strong enough to lead to a successful outcome. As Chris’ story highlights, building of trust among research partners and academics was crucial to the successful completion of the process. The importance of trust in successful PIR is often overlooked in the literature, or touched in passing (e.g. [1]). The challenges arising from the interactions with different bureaucracies made it an essential ingredient to counter the negative effects of scrutiny by officials, who did not have any previous interpersonal relationship with the research partners. Trust in the academics overseeing this process served to mitigate the anxiety around the grey areas and unknowns that research partners encountered along the way. While there are several studies highlighting the positive effects of trust on teamwork and cooperation (e.g. [11, 29]), Chris’ story also specifically pointed at one such effect, that is, the willingness to sacrifice oneself in moments of difficulty for a higher goal to be pursued as a team ([25], p. 542).

The positive aspects should however not be overemphasised: the emotional burden and the amount of time required were considerable and drained a substantial amount of human resources away from actual research activities. What emerged from the individual narratives and our collective discussions is that the pragmatic aspects of securing the different documents and “box ticking” were linked, often inseparably, with more reflexive considerations about doing health research.

#### Professionalisation through interaction with bureaucracy

The process operated as a sort of “initiation ritual” for research partners into what often appeared to them as relatively closed boundaries of the academic profession. This perception was coupled by feeling under scrutiny, explicitly articulated by Rosie, and was somewhat at odds with their previous experiences of PIR and the openness and availability that David, as the principal investigator, had put in at the early stages of research design and throughout the grant application process. David and Vito made themselves available to assist with the research passport application, not only helping with the technical aspects but also providing emotional support – as far as the constraints of their work schedules allowed. Yet, this was not always enough to counter the feeling of estrangement that Rosie, Chris and Cathy felt in different ways throughout the process. In a way, there is nothing exceptional in this situation: professional training and different stages of examination and review to obtain the appropriate qualifications and skills often put people in stressful situations (e.g. [16]). Professional researchers have to go through this at different stages of their career, and to some extent continue to do so until they retire. This trend is shaped by an environment characterised by increasing pressure to perform, partly exercised through the rapid expansion of the quantity and scope of performance review mechanisms, and a growing emphasis on continuing professional development.

By having to undergo checks on their employment experience, the demanding tasks of filling complex forms and being available for interviews with different officials, research partners were tacitly asked to behave “as if” they were professionals. This provides another angle on the debate about the professionalisation of research partners opened by a piece by Thompson et al. [48]. A widely cited rationale for PIR and patient and public involvement in other areas of health (e.g. service provision and development) is that members of the public who have used health services have “experiential expertise” [19], “experiential knowledge” [7] or “lay knowledge” [39] that is different from the expertise held by academics, and can greatly improve the results of research [45] and democratise health research in the interest of the wider public [27]. Thompson et al. [48] argue that while research partners’ experiential expertise constitutes a primary source of their contribution, this does not mean that there has to be a clear-cut separation between the professional knowledge of academic researchers and the lay knowledge of research partners. Their research shows that partners highly value professional expertise and look for ways to increase their own credibility not simply as “users”, but as knowledgeable in the disciplines guiding the research. For this reason, they refer to them as “scientifically engaged lay experts” (p. 609). In many cases research partners find themselves in a hybrid situation where they mix experiential expertise with professional expertise.

In our case, the research passports journey presumed a certain degree of professionalisation for its successful completion. The journey through the different steps became in effect a form of training into the more bureaucratic aspects of health research, also in the sense that the interpersonal interactions required of them involved a specific kind of rules and behaviors which were not common in the more open-ended processes of involvement in project team activities. The very fact that they were expected to act in a certain way when interviewed, fashion themselves with a certain degree of formality when writing their CVs and filling the forms, became an integral part of this professionalisation. This tends to reinforce Thompson et al. [48] finding that a strict demarcation between “naïve” research partners and professional experts is untenable in practice. Even when research partners are not actively seeking to professionalise themselves (as, for instance, in Rosie’s undertaking of a PhD in the field), the structures and processes at play in health research require of them some degree of professionalisation.

#### The reflexive effects of the research passports journey

The research passports application process was clearly laden with a power dynamic as structures and procedures were imposed on research partners without their active involvement in setting the policy rationale and agenda. While in practice there was a certain amount of flexibility, ultimately research partners (like any other researchers) had to comply with the requirements if they wanted to be involved in carrying out data collection in their case studies. The other side of the coin was that the process of “becoming a research partner” through interaction with the bureaucracy had important reflexive “feedback” effects on the institutional structures and the overall conduct of the research. As we mentioned in the methodology section, reflexivity is commonly used in health research to refer to awareness and reflection of one’s own position in a specific research context and the acknowledgement of “how our own personal experiences and contexts can influence, impact, and constructively inform the outcomes of any enquiry” ([40], p. 710). Sociological theories show us that structures too are reflexive, in the sense that they are engaged in a recursive movement where they constrain and enable the action of individuals and organisations, while at the same time being the outcome of these actions [18]. The agency of research partners – in alliance with the academics involved – impacted, albeit in a limited way, on the specific ways in which the overall structure of the research passports procedure unfolded. More importantly, it had a significant impact on the way research was understood and implemented within our project.

In terms of institutional context and structures, this provided an important precedent that future public involvement activities could build on, both at our university and at the NHS trusts’ level. While this might sound obvious in principle, it is important that the real bureaucratic procedures in place are not confused with statements of principle. While both the university and the NHS trusts made allowances for PIR activities, research partners acted as ground breakers. At least at the university level, this meant that it will be easier for future university-based projects to go through the same procedure. Whether this is the case for the NHS trusts, only time will tell, but we are hopeful that it was a useful learning experience for the trusts as well.

This journey had positive reflexive effects on both the research partners’ experience and views of research, and those of the academics involved. By linking together the practical realm of fulfilling requirements with their concerns and anxieties about being involved in a multi-institutional health research project, the research passports journey questioned the hidden assumptions and the often invisible rules of health research. For instance, when it came to undertaking the Good Clinical Practice certificate, it became clear that the approved course for that purpose pertained mostly to clinical trials contexts (which most of the case studies in our project were not). Even then it did not seem to provide relevant knowledge for the purposes of the project’s research. The kind of experiential knowledge that emerged from the interactions between research partners and academics questioned the usage of technical discourse to “depoliticise” (cf. [15, 35]) crucial questions about the research process, about who controls it, and for what purpose [3, 41, 50].

Another unexpected reflexive effect that emerged from the collaborative autoethnographic process was an alignment of views between research partners and Vito on the excessive use of technical discourse and bureaucratic procedures over substantive issues. Vito, as an anthropologist, was used to more flexible and reflexive forms of research, where consent procedures and permissions were more dependent on interpersonal relationships with the participants, than prolonged interactions with institutional bureaucracies.

Overall, this brand of “interpersonal reflexivity” [31] had a double effect. For research partners, it increased their understanding of research and constantly challenged them to think beyond their previous knowledge, and about their own position in the process. For the academics, this encounter with what seemed at the beginning a purely “technical” requirement, made them much more aware of the complexities and challenges of providing an inclusive and stimulating environment for PIR.

Reflexive effects have not yet received the attention they deserve in PIR debates, but there is a growing literature that acknowledges them (e.g. [19, 27, 40, 48]). Gillard et al. [19] employ a reflexive analysis to study the positive impact of the interaction with research partners on overall data quality. Read and Maslin-Prothero [40] provide a reflexive analysis of the challenges of PIR with research partners from vulnerable populations. Other work confirms our findings about the reflexive engagement of research partners with their own experience and the wider process of research ([48], p. 614; [27]). Our contribution to this body of literature is that interaction with bureaucratic and organisational dimensions which are integral to the context of research, do spur personal and interpersonal reflexive engagement. The directions the latter take can influence the overall structure and effectiveness of PIR.

A further layer of reflexivity was brought to the fore by the collaborative autoethnographic methodology we employed. The focus on individual experiences and the attempt to find common patterns and emerging themes spurred a deeper reflection about the course the project was taking. The emotional effect of reminiscing about the stress and challenges of the research passports journey led to the discussion of some of the grey areas and implicit tensions within the team. These internal team dynamics were discussed openly and in a reflexive constructive manner. The authors felt that this had a positive effect on their general sense of belonging in the project.

### Conclusion: PIR between democracy and bureaucracy

Our experiences of the research passports journey, in particular that of the research partners involved, lead to a number of practical conclusions. Project teams should allow enough time to go through all the hurdles and steps required, and they should plan in advance for the right amount of capacity needed from project leaders and administrators to support research partners through this often challenging journey. The data presented here should serve as a reminder to policy makers that the bureaucratic and organisational structures involved in PIR can sometimes produce unanticipated and unwanted negative effects on research partners, and hence on the overall quality and effectiveness of PIR. The relevant departments in universities and NHS trusts should carefully review their procedures to accommodate research partners who come from diverse background and with varying levels of professional skills. We hope that these institutions will learn from our findings and improve the research passports journey accordingly.

Beyond these practicalities and the need to enlarge the knowledge base of the bureaucratic effects of institutional processes on PIR, our discussion contributes to wider debates that are at the core of PIR normative frameworks and practices. There is a deeper tension emerging here between bureaucracy and democracy. How do we effectively channel bureaucratic structures to ensure that the democratic ideals of PIR are achieved? To what extent should we rely on bureaucracy to deliver the double goal of democratising health research and improving its quality and impact? When one looks at the wider social scientific literature on the interactions of people with different kinds of bureaucracy, our findings are not particularly surprising (e.g. [20, 49]). It is clear that bureaucracy can have, and usually does have, negative side-effects on the emotional well-being of those who go through it, and can sometimes act in exclusionary ways. In teamwork situations, it has the potential to put strain on interpersonal relationships and divert resources and focus from the substantive goals the research team has set for itself.

But we also know that bureaucracy is an essential feature of contemporary life, and we need it to ensure that democratic principles and goals are indeed applied fairly and uniformly [13]. The role of bureaucracy in the case of research passports is to ensure that quality, accountability and risk management are duly taken into account, and to ensure that procedures negotiated at different levels of involvement and consultation with different stakeholders in the wider society are thus consistently applied throughout the system. The rationale behind research passports is driven by the principles underpinning the UK Department of Health’s Research Governance Framework for Health and Social Care, revised in 2005. The overarching ethical imperative for health research is well captured by the following statement: “[t]he dignity, rights, safety and well-being of participants must be the primary consideration in any research study” [9]. We need efficient research governance to make sure that researchers – including research partners – uphold the highest ethical standards and protect research participants from harm. Checking their credentials through research passports is essential to achieve this goal.

At the same time, if we are to counter the wave of negative accounts of bureaucracy which are spreading both in academia and in general public discourse, and dispel fears of bureaucracy becoming increasingly independent from democratic accountability, we need to make sure that we strike the right balance between bureaucracy and democracy. In other words, we need to avoid the danger of PIR becoming a “technology of legitimation” of top-down managerial prerogatives (cf. [22]), and put our efforts in making PIR bureaucracy more inclusive and ultimately more democratic.

## Footnotes

1 See Wolcott [51] for a concise overview of the key features of the ethnographic method.

2 See Read and Maslin-Prothero [40] as an example of a reflexive analysis of the challenges of PIR with research partners from vulnerable populations.

3 The main difference with other collaborative autoethnographies (e.g. [17, 30]) is that we did not start from written personal narratives, but we preferred to capture these narratives through one-to-one interviews.

4 The information regarding the research passports application process presented in this section refers to procedures in place in 2012, when the research partners in the project had to obtain their research passports.

5 This is not her real name.
